# Assessment of core drug use indicators using WHO/INRUD methodology at primary healthcare centers in Bahawalpur, Pakistan

**DOI:** 10.1186/s12913-016-1932-2

**Published:** 2016-12-08

**Authors:** Muhammad Atif, Muhammad Rehan Sarwar, Muhammad Azeem, Mubeen Naz, Salma Amir, Kashaf Nazir

**Affiliations:** Department of Pharmacy, The Islamia University of Bahawalpur, Bahawalpur, Pakistan

**Keywords:** Primary healthcare center, Rational, Irrational, Use of drugs, WHO/INRUD core drug use indicators, Prescribing pattern, Patient-care, Facility-specific, Pakistan

## Abstract

**Background:**

Proper utilization of medicines is a critical component of pharmaceutical care plan. The aim of this study was to assess drug use pattern at ten primary healthcare centers (PHCCs) of the Bahawalpur district of the Punjab province of Pakistan by employing the WHO/INRUD core drug use indicators.

**Methods:**

This was a descriptive, non-experimental and cross-sectional study. For the prescribing indicators, 1000 prescriptions (100 prescriptions per PHCC) were systematically sampled out of the total 290,000 prescriptions written during January to December 2014. A total of 300 randomly selected patients (30 per PHCC) and 10 pharmacy personnel (one per PHCC) were observed and interviewed to investigate the patient-care and facility-specific indicators, respectively. We used published ideal standards for each of the WHO/INRUD indicators.

**Results:**

Among the prescribing indicators, the average number of drugs per encounter was 3.4 (SD = 0.8) (optimal range = 1.6–1.8), the drugs prescribed by the generic name were 71.6% (optimal value = 100%), the encounters with an antibiotic prescribed were 48.9% (optimal range = 20.0–26.8%), the encounters with an injection prescribed were 27.1% (optimal range = 13.4–24.1%) and the drugs prescribed from the Essential Drugs List (EDL) were 93.4% (optimal value = 100%). Among the patient-care indicators, the average consultation time was 2.2 min (SD = 0.8) (optimal value ≥10 min), the average dispensing time was 38 s (SD = 12.1) (optimal value ≥90 s), the percentage of drugs actually dispensed was 90.9% (optimal value = 100%), the percentage of drugs adequately labeled was 100% (optimal value = 100%) and the patients’ knowledge of correct dosage was 62.1% (optimal value = 100%). Among the facility-specific indicators, all PHCCs had a copy of the EDL and the key drugs available in the stock were 82% (optimal value = 100%).

**Conclusions:**

Irrational use of drugs was observed in all healthcare facilities. This study necessitates the need to implement the WHO/INRUD recommended 12 core interventions to promote rational use of medicines.

## Background

Appropriate use of medicines is essential to provide better health and medical care to patients and to the community as a whole [1]. World Health Organization (WHO) defines rational use of medicines as “patients receive medications appropriate to their clinical needs, in doses that meet their own individual requirements, for an adequate period of time, and at the lowest cost to them and their community” [2]. According to the World Bank, in developing countries, 20–50% of the healthcare expenditures are spent on medicines and other medical trifles. Studies have showed that over 50% of all medicines worldwide are prescribed or sold incorrectly and 50% of the patients are unable to use them correctly [3]. Irrational prescribing practices result in unsafe and ineffective treatment, aggravation or prolongation of disease state, harm and distress to the patients and increased costs [4, 5]. Irrational utilization of medicines can also cause an increase in morbidity and mortality associated with chronic conditions such as diabetes, hypertension, epilepsy and neurological disorders [3]. The increase in antibiotic resistance due to the overuse of antibiotics is one of the major problems of the irrational use of medicines [6, 7].

Irrational use of drugs among patients is enforcing them to lose confidence in the healthcare system. In developing countries, for example Pakistan, the problem is exacerbated by limited economic resources and lack of organized drug policy [5, 7]. The most common causes of irrational medicine use are; self-medication, polypharmacy, inappropriate use of antibiotics, overuse of injectable and prescribing of medicines which are not according to clinical practice guidelines [3, 5, 7]. Furthermore, there are numerous factors that influence irrational prescribing for example patients, practitioners/physicians, the working environment, the drug supply system, legal regulations, information and misinformation about the medicines and profit intentions from selling medicines [8–12].

The fundamental steps to limit the irrational use of medicines is to identify the type, amount and reasons of irrational use of medicines. In 1990’s, WHO in collaboration with the International Network of Rational Use of Drugs (INRUD) developed a set of indicators to measure the performance of healthcare facilities related to utilization of drugs [1]. These core drug use indicators are classified into prescribing, patient-care and facility-specific (Table 1). Assessment of drug use patterns through the WHO/INRUD indicators have been successfully implemented in more than 30 developing countries [13].

**Table 1:** Core drug use indicators and their optimal values

Core drug use indicators	Optimal values
Prescribing indicators	
Average number of medicines prescribed per patient encounter	1.6–1.8
Percent medicines prescribed by generic name	100
Percent encounters with an antibiotic prescribed	20.0–26.8
Percent encounters with an injection prescribed	13.4–24.1
Percent medicines prescribed from essential medicines list or formulary	100
Patient-care indicators	
Average consultation time (minutes)	≥10
Average dispensing time (seconds)	≥90
Percent medicines actually dispensed	100
Percent medicines adequately labeled	100
Percent patients with knowledge of correct doses	100
Facility-specific indicators	
Availability of essential medicines list or formulary to practitioners	100
Percent key medicines available	100

To-date, no study from Pakistan has reported drug use practices at the primary healthcare centers (PHCCs) based on the standard core drug use indicators. Therefore, the purpose of this study was to investigate the drug use pattern by employing the WHO/INRUD core drug use indicators at the PHCCs of the Bahawalpur district of the Southern Punjab, Pakistan. The measured values could be used as benchmarks for the healthcare facilities and as a basis for further follow-up of quality of drug use. The findings of this study will further help the policymakers to implement appropriate interventions to promote rational use of medicines.

## Methods

### Study setting

The study was conducted in the Bahawalpur district of the Punjab province of Pakistan. Bahawalpur is the 12^th^ largest city of Pakistan with an approximate population of 3,333,467 people. There are two public sector tertiary healthcare facilities in Bahawalpur. Besides this, there are 84 PHCCs in the Bahawalpur district. Out of these, 10 PHCCs were randomly selected using random number generator function. All PHCCs of Pakistan in general and the Punjab province in specific are almost similar in terms of patient mix and qualifications of the healthcare staff.

### Study design and outcome measures

A quantitative, non-experimental and cross-sectional study design was employed to evaluate the performance of PHCCs in three general areas related to rational drug use; prescribing, patient-care and facility. The optimal values for prescribing [4, 5, 7], patient-care and facility-specific indicators [5, 6, 14] were adopted from previous studies. The optimal values for consultation and dispensing time were set as ≥10 min and ≥90 s, respectively.

Zhang and Zhi developed index system for the comprehensive evaluation of healthcare system [14, 15]. This index system has been successfully employed by various healthcare systems for process improvement [14, 16]. For the calculation of indices (index of non-poly-pharmacy, index of rational antibiotic use and index of safe injection drug use) following formula was used;

$$ Index=\frac{\mathrm{Optimal}\;\mathrm{value}}{\mathrm{Observed}\ \mathrm{value}} $$

All other indices (index of generic name, index of EDL, consultation time index, dispensing time index, index of actually dispensed drugs, index of labeling of drugs, index of patients’ knowledge, index of EDL availability and index of key drugs available in stock) were calculated by the following formula;

$$ Index=\frac{\mathrm{Observed}\;\mathrm{value}}{\mathrm{Optimal}\;\mathrm{value}} $$

The optimal index for all indicators was set as 1. The values closer to 1 indicated rational drug use. The Index of Rational Drug Prescribing (IRDP) was calculated for all healthcare centers by adding the index values of all prescribing indicators [14]. Based on the IRDP values, the PHCCs were ranked from 1 to 10 (rank 1 for the higher IRDP value and rank 10 for the lower IRDP). In a similar fashion, the Index of Rational Patient-Care Drug Use (IRPCDU) and the Index of Rational Facility-Specific Drug Use (IRFSDU) were calculated. Finally, the Index of Rational Drug Supply (IRDS) was calculated for all PHCCs by adding up the total of IRDP, IRPCDU, and IRFSDU. Subsequently, the PHCCs were ranked based on the IRDS indices. The PHCC with the higher IRDS value was considered best performing PHCC in terms of rational drug use and was given rank 1.

### Data collection

The standard prescribing, patient-care and facility-specific indicator forms were used to collect the data. Reliability of the data was ensured by following the WHO guidelines and methods [1]. The data was collected during the months of January and February 2015. Trained data collectors explained the purpose of the study to the respondents and obtained their consent before data collection.

#### Prescribing indicators

For the prescribing indicators, trained data collectors randomly selected 1000 prescriptions (100 prescriptions per PHCC) out of the total 290,000 prescriptions written during the period of one year (i.e., January to December 2014). The sample was selected using systematic random sampling technique and the sampling unit was the patient encounters at each of the PHCCs [4]. To minimize the biasness arising as a consequence of seasonal alterations or discontinuation of supply cycle of the drugs, the encounters were uniformly distributed throughout the year in a way that the whole year was divided in four quarters and 25 prescriptions were taken from each quarter. The sample was limited to the encounters comprising of acute and chronic illnesses, indicating a mixture of health conditions and age of the patients. Referral or vaccination cases were excluded from the study.

#### Patient-care indicators

For the patient-care indicators, patients visiting the healthcare facilities for the diagnosis and treatment of general health problems were invited to participate in the study. A total of 300 patients (30 patients per PHCC) comprising a mixture of disease states and age groups were selected in a uniform manner throughout the clinic hours. The consented patients were observed and interviewed to obtain the required information.

#### Facility-specific indicators

In general, pharmacy personnel (not necessarily a pharmacist) dispense the drugs to the patients at the pharmacy counter of each PHCC. For the facility-specific indicators, one personnel per PHCC was selected and the consented respondents were interviewed to obtain the required information.

### Statistical analysis

Statistical Package for Social Sciences (IBM SPSS Statistics for Windows, version 21.0, Armonk, NY: IBM Corp.) was used for analysis of data. Descriptive statistics such as frequencies, percentages, mean and standard deviation were measured. Differences among the healthcare facilities were established using ANOVA test. The statistical significance was determined by *p* < .05.

**Note:** In this paper, the word ‘drug(s)’ and ‘medicine(s)’ are used interchangeably, and are similar in terms of their meaning.

## Results

### Drug prescribing indicators

The average number of drugs per encounter was 3.4 (SD = 0.8) while 71.6% (SD = 15.7) drugs were prescribed by the generic name. The percentage of encounters with an antibiotic was 48.9% (SD = 20.2). The percentage of encounters with an injection was 27.1% (SD = 9.8). The percentage of drugs prescribed from the EDL was 93.4% (SD = 7.1). The difference among the PHCCs was statistically significant for all prescribing indicators (Table 2).

**Table 2:** WHO/INRUD prescribing indicators in selected primary healthcare centers of the Bahawalpur district, Punjab, Pakistan

Primary Healthcare Centers^a^	Prescribing Indicators				
	Average number of medicines prescribed per patient encounter	Percent medicines prescribed by generic name	Percent encounters with an antibiotic prescribed	Percent encounters with an injection prescribed	Percent medicines prescribed from essential medicines list
1	3.4 (1.4)	81.2	64.0	38.0	98.5
2	3.6 (1.5)	85.1	63.0	32.0	87.6
3	3.4 (1.3)	83.0	87.0	21.0	89.2
4	3.2 (3.1)	43.7	40.0	18.0	90.7
5	3.2 (1.4)	88.5	56.0	30.0	100
6	1.9 (1.0)	50.0	31.0	25.0	100
7	5.0 (2.3)	60.0	30.0	9.0	80.5
8	4.3 (2.4)	76.0	28.0	28.0	100
9	2.9 (0.7)	66.7	29.0	43.0	100
10	3.4 (1.1)	82.0	61.0	27.0	87.9
Mean (SD)	3.4 (0.8)	71.6 (15.7)	48.9 (20.2)	27.1 (9.8)	93.4 (7.1)
ANOVA	*p* < .0005	*p* < .0005	*p* < .0005	*p* < .0005	*p* < .0005

^a^1 = Agha pur, 2 = Jamal channar, 3 = Mubarak pur, 4 = Jhangi wali, 5 = Mithra, 6 = Chak katoora, 7 = Kud wala, 8 = Khanqah sharif, 9 = Khanu wali, 10 = Kulaab

### Patient-care indicators

The average consultation time was 2.2 (SD = 0.8) minutes. The average dispensing time was 38 (SD = 12.1) seconds. 90.9% (SD = 9.5) of the prescribed drugs were actually dispensed. All dispensed drugs were adequately labeled. The percentage of patients’ knowledge of the correct dosage was 62.1 (SD = 19). The difference among the PHCCs was statistically significant for all patient-care indicators except the percentage of drugs adequately labeled (Table 3).

**Table 3:** WHO/INRUD patient-care and facility-specific indicators in selected primary healthcare centers of the Bahawalpur district, Punjab, Pakistan

Primary Healthcare Centers	Patient-Care Indicators					Facility-Specific Indicators	
	Average consultation time (minutes)	Average dispensing time (seconds)	Percent medicines actually dispensed	Percent medicines adequately labeled	Percent patients with knowledge of correct doses	Availability of essential medicines list to practitioners	Percent key medicines available
1	2.3 (1.5)	43.1 (34.7)	87.3	100.0	67.0	100.0	90.0
2	2.5 (1.2)	43.0 (17.1)	91.1	100.0	77.0	100.0	70.0
3	2.4 (1.0)	36.7 (10.8)	91.2	100.0	77.0	100.0	80.0
4	0.7 (0.4)	15.5 (7.7)	100.0	100.0	30.0	100.0	90.0
5	2.1 (0.8)	42.6 (16)	85.8	100.0	67.0	100.0	80.0
6	1.3 (0.6)	31.3 (14)	68.3	100.0	33.0	100.0	80.0
7	2.9 (1.4)	30.9 (10.5)	100.0	100.0	53.0	100.0	70.0
8	2.1 (1.2)	36.9 (25.8)	91.5	100.0	64.0	100.0	90.0
9	3.6 (1.1)	63.3 (50.7)	100.0	100.0	90.0	100.0	90.0
10	2.1 (1.0)	37.1 (20.6)	93.6	100.0	63.0	100.0	80.0
Mean (SD)	2.2 (0.8)	38.0 (12.1)	90.9 (9.5)	100.0	62.1 (19)	100.0	82.0 (7.9)
ANOVA	*p* < .0005	*p* < .0005	*p* < .0005	------^a^	*p* < .0005	------^a^	*p* < .0005

^a^ANOVA was not applied for these indicators as there was no variation in their values

### Facility-specific indicators

The percentage availability of the EDL copy was 100% (SD = 0.0) and of key drugs in the stock was 82% (SD = 7.9). Among the PHCCs, there was a significant difference for all facility-specific indicators except the percentage availability of the EDL copy (Table 3).

The IRDP values showed that the PHCC number six was performing well with regard to rational prescribing practices. Similarly, the IRPCDU values indicated that the PHCC number nine showed better results among all PHCCs with regard to patient-care indicators. The PHCC number one, four, eight and nine showed comparable better results with regard to facility-specific indicators. Overall, the IRDS values showed that the PHCC number nine was performing well among other PHCCs in terms of rational drug use (Table 4).

**Table 4:** Performance indicators for selected primary healthcare centers of the Bahawalpur district, Punjab, Pakistan

Performance Indicators	Primary Healthcare Centers									
	1	2	3	4	5	6	7	8	9	10
Prescribing Indicators										
(1) Non-polypharmacy index	0.53	0.5	0.53	0.56	0.56	0.95	0.36	0.42	0.62	0.53
(2) Generic name index	0.81	0.85	0.83	0.44	0.88	0.50	0.60	0.76	0.67	0.82
(3) Rational antibiotic index	0.42	0.43	0.31	0.67	0.48	0.86	0.89	0.96	0.92	0.44
(4) Injection safety index	0.63	0.75	1.0	1.0	0.80	0.96	1.0	0.86	0.56	0.89
(5) Essential drugs list index	0.99	0.88	0.89	0.91	1.0	1.0	0.81	1.0	1.0	0.88
IRDP	3.38	3.41	3.56	3.58	3.72	4.27	3.66	4.0	3.77	3.56
Rank	9	8	7	6	4	1	5	2	3	7
Patient-Care Indicators										
(6) Consultation time index	0.23	0.25	0.24	0.07	0.21	0.13	0.29	0.21	0.36	0.21
(7) Dispensing time index	0.48	0.48	0.41	0.17	0.47	0.35	0.34	0.41	0.70	0.41
(8) Dispensed drugs index	0.87	0.91	0.91	1.0	0.85	0.68	1.0	0.91	1.0	0.93
(9) Labeled drugs index	1.0	1.0	1.0	1.0	1.0	1.0	1.0	1.0	1.0	1.0
(10) Patients’ knowledge index	0.67	0.77	0.77	0.30	0.67	0.33	0.53	0.64	0.90	0.63
IRPCDU	3.25	3.41	3.33	2.54	3.20	2.49	3.16	3.17	3.96	3.17
Rank	4	2	3	8	5	9	7	6	1	6
Facility-Specific Indicators										
(11) Index of EDL	1.0	1.0	1.0	1.0	1.0	1.0	1.0	1.0	1.0	1.0
(12) Index of key drugs in stock	0.90	0.70	0.80	0.90	0.80	0.80	0.70	0.90	0.90	0.80
IRFSDU	1.90	1.70	1.80	1.90	1.80	1.80	1.70	1.90	1.90	1.80
Rank	1	3	2	1	2	2	3	1	1	2
Grand total										
IRDS	8.53	8.52	8.69	8.02	8.72	8.56	8.52	9.07	9.63	8.53
Rank	6	7	4	8	3	5	7	2	1	6

Optimal index = 1, *IRDP* Index of Rational Drug Prescribing, *IRPCDU* Index of Rational Patient-Care Drug Use, *EDL* Essential Drugs List, *IRFSDU* Index of Rational Facility-Specific Drug Use, *IRDS* Index of Rational Drug Supply

## Discussion

The irrational prescribing practices exist all over the world and ultimately lead to unwanted effects in patients [6]. In this study, the WHO/INRUD drug use indicators were used to identify current treatment practices that may help to resolve problems regarding drug therapy. Our findings would also be a source of baseline information for continuous monitoring of drug therapy.

A number of developing and transitional countries have conducted similar studies. Ten previously published studies from different countries were reviewed and included for comparison purposes (Table 5).

**Table 5:** Results of WHO/INRUD Indicator studies in different countries

Performance Indicators	Study Reference										Mean
	[54]	[14, 58]	[55]	[6]	[41]	[28]	[48]	[42]	[36]	[49]	
Average number of medicines prescribed per patient encounter	2.2	2.4	2.3	2.5	2.3	3.0	2.2	2.2	2.4	3.1	2.5
Percent medicines prescribed by generic name	74.0	61.2	5.1	95.4	75.5	63.1	99.0	79.4	99.8	10.1	66.3
Percent encounters with an antibiotic prescribed	37.0	32.2	60.9	39.2	35.4	54.2	43.0	24.9	66.0	33.0	42.6
Percent encounters with an injection prescribed	11.0	2.0	1.2	9.9	19.0	38.0	18.0	10.6	2.4	2.4	11.4
Percent medicines prescribed from EML	78.0	99.2	93.0	95.4	87.1	75.6	98.8	90.3	99.7	65.2	88.2
Average consultation time (minutes)	5.8	7.3	3.9	7.1	3.6	6.1	3.7	6.2	4.4	2.3	5.0
Average dispensing time (seconds)	17.0	100	28.8	47.4	39.9	18.1	37.0	78.0	234	258	85.8
Percent medicines actually dispensed	66.0	99.6	81.8	95.9	91.6	99.1	84.5	83.4	100	81.0	88.3
Percent medicines adequately labeled	63.0	10.0	91.4	0.0	87.6	55.9	86.2	70.1	0.0	99.4	56.4
Percent patients with knowledge of correct doses	54.0	79.3	77.7	94.0	96.1	86.5	81.7	72.1	55.0	74.3	77.1
Availability of EML to practitioners	50.0	90.0	100	80.0	100	100	100	50.0	100	100	87
Percent key medicines available	55.0	59.2	80.0	78.3	100	91.7	86.5	65.0	86.6	84.0	78.6

*EML* Essential Medicines List; Study number 29 was in Brazil; 14,30 in Saudi Arabia; 31in Jordan; 6 in Egypt; 32 in Tanzania; 28 in Swaziland; 33 in Mozambique; 34 in Ethiopia; 35 in Cambodia; 36 in India

### Prescribing indicators

A prescription is the reflection of prescribers’ attitude towards the disease being treated and type of healthcare system in the country. The results of this study revealed that the average number of drugs per encounter was 3.4 (SD = 0.8), and the difference among the PHCCs was statistically significant (*p* ≤ 0.0005). Our value is higher than the admissible range of 1.6–1.8 drugs per encounter. In contrast to our findings, the average number of drugs per encounter was lower in a majority of developing countries (range = 1.3–3.0). For example, this value was 3.0 in Sri Lanka [17], 2.1 in Nepal [18], 2.2 in Vietnam [19], 2.3 in Botswana [20], 2.3 in Burkina Faso [21], 1.8 in Malawi [22], 1.4 in Sudan [23] and 1.3 in Zimbabwe [24]. However, the average number of drugs per encounter was higher in Afghanistan (3.9) [25], India (5.6) [26], Ghana (4.8) [27] and Nigeria (5.2) [28]. Several reasons might be responsible for higher number of drugs in a prescription. For example, incompetency on the part of physicians, unavailability of clinical practice guidelines, financial incentives to the prescribers, lack of continuous medical education of the prescribers and the shortage of therapeutically correct drugs [5]. Polypharmacy can adversely influence the treatment outcomes as the patients are more likely to be non-compliant and are at higher risk of adverse events. Moreover, unnecessarily prescribed medicines may lead to fiscal implications for national healthcare systems [5, 7].

There are high recommendations of WHO for generic prescription [29]. WHO deems it as a safety measure for patients as it clearly depicts and gives easy accessible information, and leads to better communication among healthcare providers [6]. The results revealed that the percentage of drugs prescribed by the generic name was 71.6% (SD = 15.7). The difference among the PHCCs was statistically significant (*p* ≤ 0.0005) (Table 2). The proposed optimal value of drugs prescribed by the generic name is 100% (Table 1). In a majority of developing countries, this value was <40%, for example, in Andorra 6% [30], Uzbekistan 38.3% [31], Ecuador 37% [24], Yemen 39.2% [32], Palestine 5.5% [33], Lebanon 2.9% [34] and India 11.5% [35]. In a few countries, this value was near the optimal level as reported in Cambodia (99.8%) [36], Timor-Leste (92%) [37] and Ethiopia (98.7%) [4]. Discrepancy among the findings of various studies might be associated with several reasons such as faith of prescribers on branded products, extensive promotional activities of pharmaceutical companies influencing prescribers’ decisions or lack of legal binding to prescribe generic medicines.

The results showed that the percentage of encounters with an antibiotic prescribed was 48.9% and the difference among the PHCCs was statistically significant (*p* ≤ 0.0005). The proposed optimal range for an antibiotic prescribed is 20.0–26.8% (Table 1). In a majority of developing countries, the percentage of encounters with an antibiotic prescribed lies between 24% and 50% as in Bangladesh 25% [38], India 44.8% [39], Nepal 43% [18], Lao People’s 47% [40], Burkina Faso 33.1% [21], Burundi 50% [40], Tanzania 35.4% [41], Brazil 28.8% [40] and Ethiopia 24.9% [42]. In a few countries, this value was much higher for example in Timor-Leste 70% [37], Burma 76% [43], Kenya 73.4% [40] and Sudan 70.4% [40]. Irrational prescription of antibiotics is a universal problem that ultimately leads to adverse drug reactions and frequent hospital admissions [6]. Overuse and misuse of antibiotics is alarming situation regarding population health, especially in developing countries. In our setting, there was lack of laboratory facilities to perform microbiological testing. This could affect prescribers’ behaviour, and consequently they may have increased tendency to prescribe broad spectrum antibiotics to cover suspected infections. Unavailability of clinical practice guidelines, incompetency of the physicians and cultural beliefs in a community could be a few other reasons associated with over prescribing of antibiotics.

The present study revealed that the percentage of encounters with an injection prescribed was 27.1% and the difference among the PHCCs was significant (*p* ≤ 0.0005) (Table 2). The proposed optimal range for an injection prescribed is 13.4–24.1% (Table 1). In contrast to our findings, the percentage of encounters with an injection prescribed was 17% in Afghanistan [25], 18% in Lao People’s [44], 9% in Botswana [20], 10% in Burundi [40] and 9.1% in Kuwait [45]. However, in some countries, the percentage of encounters with an injection prescribed was even higher than our findings for example, 45.8% in India [35], 57.6% in Cambodia [40], 41.8% in Cameroon [46] and 80% in Ghana [27]. An excessive use of injections, in case appropriate oral dosage forms are available, may lead to higher probability of blood borne diseases [3]. Moreover, injections are always costlier than the oral formulations [6]. Limited availability of alternative modes of therapy for young children, and attitudes and beliefs of healthcare providers and patients are a few reasons associated with the use of injectable. In Pakistani rural culture, patients sometimes themselves compel the physicians to prescribe injectable with a belief that this dosage form provides quick and complete relief. The percentage of drugs prescribed from the EDL/formulary was 93.4% and the difference among the PHCCs was significant (*p* ≤ 0.0005) (Table 2). The proposed optimal value for the percentage of drugs prescribed from the EDL/formulary is 100% (Table 1). The findings of our study are comparable to other studies conducted in China (95%) [47], Bangladesh (85%) [38], Nepal (86%) [18], Burma (94.8%) [43], Mali (94.6%) [40], Mozambique (98.8%) [48] and Colombia (94.2%) [40]. Rational prescribing means to prescribe drugs from the EDL issued by WHO because medicines in EDL are older, already tested in practice with established clinical use, and are of lower cost than the newer drugs [6]. However, physicians may choose non EDL drugs due to inadequate supply of the EDL drugs.

### Patient-care indicators

The results of current study demonstrated that the average consultation time was 2.2 min (optimal time ≥ 10 min), and the difference among the PHCCs was statistically significant (*p* ≤ 0.0005) (Table 3). The short consultation time reported in our study could be correlated with a large number of patients per physician. Such a short consultation time has correspondence with the values measured in other developing countries for example, Malawi 2.5 min [22], India 2.3 min [49], Bangladesh 1.0 min [24], Indonesia 3.0 min [24], Ethiopia 2.9 min [50] and Kuwait 2.8 min [45]. However, there are certain studies conducted in China (9.5 min) [51], Nigeria (11.3 min) [52] and Sweden (22.5 min) [53] which reported better consultation time. According to WHO, insufficient consultation time leads to incomplete patient examination and subsequent irrational therapy [6]. Consultation time within optimal range is considered sufficient for proper history taking, complete physical examination, appropriate health education and a good physician–patient interaction. Short consultation time could be the consequence of increased work load of the physicians, and ethnic, religious or socioeconomic barriers between patients and healthcare providers.

The study reported an average dispensing time of 38 s (optimal value ≥90 s). The difference among the PHCCs was statistically significant (*p* ≤ 0.0005) (Table 3). This finding is almost comparable to the findings from Brazil (17 s) [54], Jordan (28.8 s) [55], Swaziland (18.1 s) [28], Nigeria (12.5 s) [56], Mozambique (37 s) [48], China (25 s) [57] and Bangladesh (23 s) [38]. However, dispensing time was higher in Nepal (86.1 s) [18], Ethiopia (78 s) [42] and Saudi Arabia (99.6 s) [58]. Shorter dispensing time is insufficient for adequate labeling, and to provide complete information about drug regimen, un-wanted drug effects and precautions of drugs. Insufficient information about therapy could lead to non-compliance and subsequent adverse events. In our setting, shorter dispensing time was related to higher patient load. Moreover, as the pharmacy staff was non-pharmacist, therefore, they had limited scope to counsel the patients which resulted in a shorter dispensing time.

The percentage of drugs actually dispensed was 90.9% (optimal value 100%) and the difference among the PHCCs was statistically significant (*p* ≤ 0.0005). Our value is higher than reported in Brazil (66%) [54], Jordan (81.8%) [55], Tanzania (56.2%) [59], Nigeria (85.3%) [52], Ethiopia (83.4%) [42] and Nepal (83%) [18]. However, the studies from Saudi Arabia (99.6%) [14], Kuwait (97.9%) [45], Egypt (95.9%) [6], Swaziland (99.1%) [28] and Niger (100%) [60] reported even higher values. The main reason involved in the low percentage of actually dispensed drugs could be inadequate availability of drugs in the stock. WHO recommends that dispensed drugs should be adequately labeled with respect to patient’s name, dose of the drug and regimen [1]. Our findings revealed that drug labeling practice was 100% which is good omen. In Saudi Arabia, it represented 10% [58], in China 95% [57], in Cambodia 0.0% [36], in Swaziland 55.9% [28], in Tanzania 20.1% [59] and in Kuwait it was 66.9% [45].

According to our findings, patient’s knowledge of correct dose was 62.1% (optimal value 100%). It was higher than that reported in Brazil (54%) [54], Kuwait (26.9%) [45], Tanzania (37.9%) [59], Malawi (27%) [22] and Cambodia (55%) [36]. However, relatively better values were reported in studies from Bangladesh (82%) [24], Nigeria (93%) [52] and Egypt (94%) [6]. Patient’s knowledge about correct dosage is highly significant to avoid over use and abuse of drugs, and prevent adverse events that ultimately affect patient’s health. As per our understanding, limited drug-related knowledge of the patients could be associated with increased workload of the healthcare providers, poor understanding skills of the patients and unavailability of qualified pharmacists at the pharmacies.

### Facility-specific indicators

The study revealed that all PHCCs had a copy of EDL/formulary which is in line with the proposed norms (optimal value 100%). However, the percentage of key drugs in the stock were 82% (optimal value 100%). Limited availability of key drugs might be associated with budgetary constraints, inadequate drug supply system or poor inventory management of the responsible staff. Contrary to our findings, studies from Saudi Arabia (90%) [58], Brazil (50%) [54], Egypt (80%) [6], Ethiopia (50%) [42] and Bangladesh (16%) [38] reported that a copy of EDL was not available at all healthcare centers. Similarly, with regard to percentage availability of key drugs in stock, studies from Brazil (55%) [54], Saudi Arabia (59.2%) [58], Nigeria (62%) [56], Malawi (67%) [22], Ethiopia (65%) [42] and Bangladesh (54%) [38] showed lower values. However, findings from Jordan (80%) [55] and Egypt (78.3%) [6] were almost comparable to our findings. Some countries even reported better values for the percentage availability of key drugs in stock for example, Tanzania (100%) [41], Swaziland (91.7%) [28], Nigeria (90.9%) [28], Cambodia (86.6%) [36] and Nepal (90%) [18]. Shortage of the essential drugs is disadvantageous for patients in terms of their health and out-of-pocket expenditures [6]. To ensure proper healthcare, WHO recommends that physicians should be adherent to the drugs listed in the EDL/formulary while prescribing.

## Conclusion and recommendations

This study demonstrated irrational drug use practices in all healthcare facilities. The observed values for all INRUD indicators deviated from the established norms. However, medicines were adequately labeled and the EDL was available at all healthcare facilities. Based on these findings, it is recommended that there should be continuous education and training of physicians about rational prescribing of antibiotics and injections. Physicians should also be motivated to enhance generic prescribing and prescribing from the EDL/Formulary. Patient-to-physician ratio should be decreased for the prolongation of consultation time, which allows thorough history taking, comprehensive examination and development of therapeutic relationship between patient and physician. Further, we recommend that pharmacists should be appointed at all PHCCs for proper dispensing of medicines and improvement of patients’ knowledge about drugs. Availability of key drugs in stock should be improved to ensure effective treatment of general health-related problems.

### Limitations

These findings could not be generalized for Pakistan. However, based on the fact that a uniform healthcare policy is implemented throughout, and medical graduates from various institutions are working in the Bahawalpur district, the practices could be assumed almost similar to other districts of Pakistan. In this study, we did not find the reasons of irrational drug use, which could be considered in future studies.

## Abbreviations

EDL: Essential drugs list
EML: Essential medicines list
INRUD: International network for the rational use of drugs
IRDP: Index of Rational Drug Prescribing
IRDS: Index of Rational Drug Supply
IRFSDU: Index of Rational Facility-Specific Drug Use
IRPCDU: Index of Rational Patient-Care Drug Use
PHCC: Primary Healthcare Center
SPSS: Statistical Package for Social Sciences
WHO: World Health Organization
